# Still in the closet: the invisible minority in medical education

**DOI:** 10.1186/1472-6920-14-171

**Published:** 2014-08-15

**Authors:** Jessica Lapinski, Patricia Sexton

**Affiliations:** 1 A.T. Still University, Kirksville College of Osteopathic Medicine, Kirksville, Missouri, USA

**Keywords:** LGB osteopathic medical students, Cultural competency, Mental health, LGBT campus climate

## Abstract

**Background:**

To investigate the relationship between sexual orientation and gender identity in regard to levels of depression; levels of perceived social support; comfort with disclosure of orientation; and the lesbian, gay, bisexual, and transgender (LGBT) campus climate.

**Methods:**

E-mail invitations to participate in the current cross-sectional questionnaire-based study were sent to all thirty US osteopathic medical schools in August 2012; six schools responded and disseminated the survey to their students. Participating students completed an anonymous web-based survey, and informed consent was obtained when they accessed the survey. The survey was designed specifically for the current study but contained scales used with permission from previously published research. Analysis procedures included nonparametric tests, one-way analysis of variance and Pearson’s correlations.

**Results:**

Of the 4112 students invited to participate in the survey, 1334 (32.4%) completed it. Approximately 85% of respondents self-identified as heterosexual only. No respondents identified as transgender. In general, LGB students indicated higher levels of depression (*P* < .001), slightly lower levels of perceived social support (*P* < .001), and more discomfort with disclosure of sexual orientation (*P* < .001). A majority of students rated their campus climate as noninclusive.

**Conclusions:**

Results of the current study indicated a relationship between sexual orientation and depression, perceived social support, comfort with disclosure of orientation, and the LGBT campus climate in osteopathic medical students. In the future, osteopathic medical schools should consider closely examining their campus culture in order to create a more positive and inclusive environment for all its students.

## Background

Lesbian, gay, bisexual, and transgender (LGBT) individuals are part of a minority that is often overlooked and stigmatized [1-3]. As such, these individuals are subject to a multitude of stressors [4]. Research suggests that negative experiences resulting from LGBT stigma can lead to chronic stress and emotional distress among LGBT adolescents and adults [5-8] and that emotional distress is associated with higher rates of depression, psychiatric disorders, suicide attempts, and substance abuse [9-11]. Research also suggests a relationship between emotional distress and perceived LGBT discrimination [12]. As a result, LGBT acceptance may be directly related to the academic success of LGBT students [13]. Many LGBT students find it difficult to focus on academics because of stigmatization, intolerance, chronic stress, and discrimination [13,14]. For medical students, academic success is crucial to establishing a baseline for effective patient care, and evidence suggests that LGBT medical students experience a variety of barriers as they progress through the medical education system [15-17].

Many LGBT medical students report discomfort with disclosure of their sexual orientation because of fear of negative reactions from classmates and professors [15]. In a survey by Merchant et al [18], 95% of medical school applicants did not disclose their orientation, of which 15% feared that disclosure would result in rejection. In another study, LGBT students expressed concern that disclosure of their sexual orientation could lead to negative evaluation [15], an outcome supported by other studies [19,20]. In a study by Schatz and O’Hanlan [19], lesbian, gay, and bisexual physicians reported discrimination in the workplace based on their sexual orientation, including denial of referral and promotions, refusal of privileges, and verbal harassment. A more recent study [20] examining the same variables found decreased but similar discriminatory behaviors. Both studies reported high rates of derogatory comments, substandard treatment, and disrespect toward LGBT patients and coworkers [19,20]. In another study, lesbian physicians were 4 times more likely than heterosexual female physicians to report experiencing harassment because of their sexual orientation, particularly during the training years [21]. Given these findings [19-21], it is not surprising that LGBT students consider the presence of identifiable supports, inclusive LGBT-curricula, and effective nondiscrimination policies before disclosing their orientation [15].

In the osteopathic medical profession, students are taught to use a holistic approach in the practice of medicine, incorporating aspects of mind, body, and spirit into patient care [22]. Issues of diversity and the intricacies of competently treating patients from diverse backgrounds are usually contained within a holistic approach. Therefore, surveying members of the osteopathic medical profession may be appropriate for studying LGBT awareness and acceptance and for investigating if an LGBT friendly environment improves the educational outcomes of LGBT medical students and the treatment of LGBT patients.

The current study was part of a larger study that investigated LGBT acceptance in a sample of students attending osteopathic medical schools. The purpose of the current study was to investigate the relationship between sexual orientation and gender identity in regard to levels of depression, levels of perceived social support, comfort with disclosure of orientation, and the LGBT campus climate. We hypothesized that self-identified LGB students and transgender students would report lower levels of social support and higher levels of depression and discomfort with disclosure of their orientation. In addition, we hypothesized that LGBT students would be more likely to rate the campus climate as noninclusive in regard to LGBT issues.

## Methods

The data from the current cross-sectional questionnaire-based study were collected from an anonymous, self-completed, web-based survey (SurveyMonkey, Inc.) completed by medical students recruited from US osteopathic medical schools. An e-mail invitation to participate in the study was sent to the deans’ offices and academic affairs offices of all thirty US osteopathic medical schools. The invitation contained an overview of the study and a survey link, which the school was requested to disseminate. Six schools agreed to participate in the study and disseminate the survey to their students. The sole inclusion criterion was current enrollment in an osteopathic medical school. Students were given an electronic informed consent before entering the survey, and by clicking the "Next page" button, participants gave their consent to participate in the study. Upon completion of the survey, participants were compensated with a $5 Amazon.com gift card, regardless of whether they fully completed the survey. Ethical approval was obtained from the A.T. Still University Institutional Review Board-Kirksville.

### Study survey

The survey used in the current study was created specifically for the study and was composed of standard scales that have been used in multiple studies [23-25]. Basic demographic information was collected and consisted of gender, age, race, and religion. Students were also asked to identify which osteopathic medical school they attended and their current year in medical school.

The Klein Sexual Orientation scale was included in the study survey to determine the sexual orientation of respondents [23]. This scale examines various aspects of sexual identity as they relate to the respondent’s past, current, and ideal sexual history [23]. Respondents are allowed to rate their identity on a scale of 1 to 7, with 1 being heterosexual only and 7 being homosexual only. For the current study, we only used the participant’s current self-identification, and categorized participants as either LGB or heterosexual only.

The Major Depression Inventory (MDI) [24] was also included in the study survey and is a ten item, self-report questionnaire that measures levels of depression. It covers the ten International Classification of Diseases 10th edition symptoms of depression, which are equivalent to those included in the Diagnostic and Statistical Manual of Mental Disorders 4th edition [24]. The MDI uses a 6-point Likert scale (1 = All the time, 6 = At no time) to determine how much time the respondent has exhibited a given symptom in the past 2 weeks [24]. Raw scores range from 0 to 50, with scores divided into categories measuring the severity of depression. Mild depression has an MDI range from 20 to 24, moderate depression from 25 to 29, and severe depression from 30 to 50 [24].

The perceived social support scale used in the study survey was a modified six item scale that examined the extent to which an individual felt cared for [25]. The comfort with disclosure of orientation scale was a nine item scale that examined the degree to which individuals felt comfortable being open about their sexuality. Responses to both scales were measured using a 5-point Likert scale (1 = Strongly Disagree, 5 = Strongly Agree).

The LGBT-Friendly Campus Climate Score [26] was included in the study survey and examined the extent to which a campus had LGBT-friendly policies, programs, and practices. The scale was modified with permission and included 5 of the 8 LGBT-friendly factors included in the original scale—policy inclusion, support and institutional commitment, academic life, student life, and counseling and health. LGBT policy inclusion included five items that examined the extent to which the institution had inclusive nondiscrimination policies. LGBT support and institutional commitment included three items that examined the availability of staff and resources to support LGBT students. LGBT academic life included two items that asked the extent to which LGBT issues were integrated in the curriculum. LGBT student life included six items that examined the availability of social and academically oriented clubs that focused on LGBT issues. LGBT counseling and health included three items that examined the availability of medical and psychosocial support for LGBT students. Each of the 5 factors was weighted and summed, resulting in scores that ranged from 0 to 50. This range was converted to a 5-star system with the following cut-off points in place: 45 through 50 = 5 stars, 40 through 44 = 4.5 stars, 35 through 39 = 4 stars, 30 through 34 = 3.5 stars, 25 through 29 = 3 stars, 20 through 24 = 2.5 stars, 15 through 19 = 2 stars, 10 through 14 = 1.5 stars, and 0 through 9 = 1 star [26].

### Statistical analysis

Because data were not normally distributed, analyses examining levels of depression, levels of perceived social support, and comfort with disclosure of orientation used nonparametric tests, mainly the Mann Whitney and Kruskall Wallis tests. The median score, as well as the interquartile range (IQR) is presented. The LGBT-Friendly Campus Climate Score was examined using one-way analysis of variance. Pearson’s correlations were used to examine relationships between levels of depression, levels of perceived social support, comfort with disclosure of orientation, and the LGBT-Friendly Campus Climate Score.

A Cronbach’s test was used to measure the internal consistency of our survey scales. The MDI had a Cronbach α of .92. The perceived social support scale had a Cronbach α of .94. The comfort with disclosure of orientation scale had a Cronbach α of .71. The LGBT-friendly Campus Climate Score had a Cronbach α of .89.

Data were analyzed using the Statistical Package for Social Sciences (SPSS, Chicago, IL). A *P* value of .01 was considered significant.

## Results

### Respondent characteristics

Six US osteopathic medical schools agreed to participate in the study resulting in a total sample size of 4112 students. A total of 1698 participants entered and started the online survey, resulting in a 41.3% response rate. From this total, 363 participants dropped out, with the majority prematurely exiting the survey once they reached the Klein Sexual Orientation scale. Therefore, 1334 participants completed the entire survey, resulting in a 32.4% completion rate. There were no significant participant characteristics (gender, age, race, religion, school attended, or year in school) that predicted failure to complete the survey.

Table 1 displays the demographic characteristics of the survey respondents. About equal numbers of male (n = 628) and female (n = 706) osteopathic medical students responded to the survey. Most respondents were aged between 18 and 35 years and were white and Christian. A slightly higher proportion of first-year (n = 410) and second-year (n = 394) medical students responded. Approximately 85% of respondents self-identified as heterosexual only, with the remainder of respondents self-identifying somewhere within the LGB spectrum. No participants identified as transgender, making it impossible to conduct any analysis of this group.

**Table 1:** Demographic characteristics of survey respondents

**Characteristic**	**No. (%)**
Gender	
Male	628 (47.1)
Female	706 (52.9)
Transgender	0 (0)
NA	1 (0.1)
Age	
Under 18	1 (0.1)
18–25	611 (45.8)
26–35	669 (50.1)
36–45	48 (3.6)
46–55	6 (0.4)
Over 55	0 (0)
Race	
White	1047 (78.4)
Nonwhite	256 (19.2)
NA	32 (2.4)
Religion	
Christian	843 (63.1)
No religion	234 (17.5)
Personal spirituality	117 (8.8)
Jewish	30 (2.2)
Other	28 (2.1)
NA	26 (1.9)
Buddhist	24 (1.8)
Muslim	18 (1.3)
Hindu	15 (1.1)
School attended	
Des Moines University College of Osteopathic Medicine	422 (31.5)
Western University of Health Sciences College of Osteopathic Medicine of the Pacific	261 (19.6)
A.T. Still University Kirksville College of Osteopathic Medicine	249 (18.7)
A.T. Still University School of Osteopathic Medicine in Arizona	163 (12.2)
Rocky Vista University College of Osteopathic Medicine	123 (9.2)
University of Pikeville-Kentucky College of Osteopathic Medicine	106 (7.9)
NA	11 (0.8)
Year in school	
1st year	410 (30.7)
2nd year	394 (29.5)
3rd year	272 (20.4)
4th year	253 (19.0)
NA	6 (0.4)
Self-identified sexual orientation	
Heterosexual only	1135 (85.0)
Heterosexual mostly	115 (8.6)
Heterosexual/gay-lesbian equally	19 (1.4)
Gay/lesbian mostly	25 (1.9)
Gay/lesbian only	41 (3.1)

*Abbreviation:NA* not applicable or students who chose "Prefer not to answer".

### Sexual orientation comparisons

**Figure 1:**
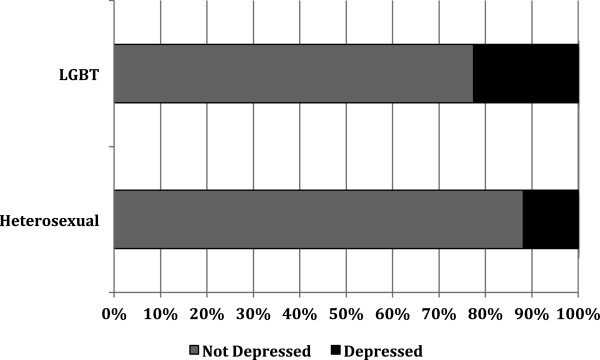
Levels of depression for participants by sexual orientation.

When examining levels of depression, respondents had a median score of 8 (IQR = 5–13). LGB students indicated higher levels of depression than heterosexual only students (*z* = -3.7*, P <* .000) (Figure 1). In general, 22.5% of LGB students and 11.8% of heterosexual only students met the clinical criteria for depression, making the odds of LGB students being depressed 2.2 times greater than heterosexual only students (95% CI, 1.5 to 3.2; *P =* .0001).

All students reported high positive levels of perceived social support with a median score of 5 (IQR = 4.8–5.0) (Table 2). However, LGB students were slightly more likely to report lower levels of support than heterosexual only students (*z* = -4.4, *P =* .001, Cohen’s d = 0.21).

**Table 2:** Participant responses for the perceived social support scale

**Question**		**Response, No. (%)**					**Mean (SD)**
		**Strongly disagree**	**Somewhat disagree**	**Neutral**	**Somewhat agree**	**Strongly agree**	
The people close to me let me know that they care about me	Hetero	13 (1.2)	11 (1.0)	16 (1.3)	214 (19.0)	875 (77.5)	4.7 (.66)
	LGBT	1 (0.5)	3 (1.5)	1 (0.5)	61 (30.8)	132 (66.7)	4.6 (.63)
I have someone in whose opinions I have confidence	Hetero	12 (1.1)	3 (0.3)	19 (1.7)	110 (9.8)	981 (87.2)	4.8 (.57)
	LGBT	1 (0.5)	1 (0.5)	2 (1.0)	37 (18.7)	157 (79.3)	4.8 (.54)
I have someone that I feel I can trust completely	Hetero	13 (1.2)	15 (1.3)	13 (1.2)	86 (7.6)	999 (88.7)	4.8 (.63)
	LGBT	3 (1.5)	3 (1.5)	5 (2.6)	41 (20.7)	146 (73.7)	4.6 (.75)
I have people around me who will help me to keep my spirits up	Hetero	12 (1.1)	4 (0.4)	16 (1.4)	140 (12.4)	954 (84.7)	4.8 (.58)
	LGBT	1 (0.5)	1 (0.5)	4 (2.0)	48 (24.3)	144 (72.7)	4.7 (.59)
There are people in my life who make me feel good about myself	Hetero	10 (0.9)	7 (0.6)	13 (1.2)	113 (10.0)	982 (87.3)	4.8 (.56)
	LGBT	1 (0.5)	0 (0)	5 (2.6)	46 (23.2)	146 (73.7)	4.7 (.57)
I have at least one friend or relative I want to be with when I am feeling down or discouraged	Hetero	13 (1.2)	7 (0.6)	33 (2.9)	86 (7.6)	986 (87.7)	4.8 (.63)
	LGBT	1 (0.5)	7 (3.5)	5 (2.6)	30 (15.2)	155 (78.2)	4.7 (.74)

**Figure 2:**
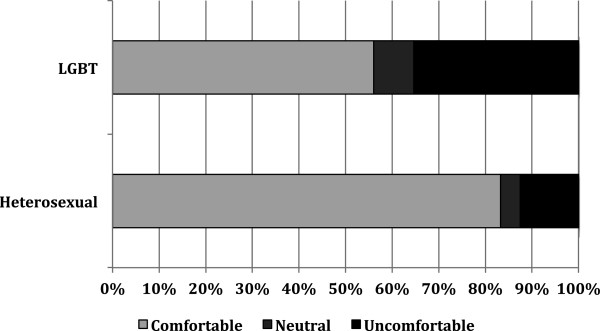
Comfort with disclosure of orientation for participants by sexual orientation.

When examining comfort with disclosure of orientation, respondents had a median score of 2.4 (IQR = 2.0–2.9). LGB students were more likely to report feeling uncomfortable with disclosure of sexual orientation (*z* = -9.0, *P <* .000) (Figure 2). In general, 43.9% of LGB students and 16.7% of heterosexual only students reported discomfort with disclosure, making the odds of LGB students having discomfort with disclosure 3.9 times greater than heterosexual only students (95% CI, 2.8 to 5.4; *P <* .000).

Finally, when examining the LGBT-friendly Campus Climate Score a majority of students (66.1%) rated their campus with 3 stars or less. LGB students were more likely to rate the campus climate as noninclusive in regard to LGBT issues (*z* = -2.3, *P <* .000, Cohen’s d = 0.39) (Table 3).

**Table 3:** Participant responses to the Lesbian, Gay, Bisexual, and Transgender (LGBT) campus climate scale

**Question**		**Response, no. (%)**		
		**Yes**	**Do not know**	**No**
**LGBT Policy Inclusion**				
Does your campus prohibit discrimination based on sexual orientation by including the words "sexual orientation" in its primary non-discrimination statement or Equal Employment Opportunity policy?	Hetero	672 (62.1)	80 (7.4)	330 (30.5)
	LGBT	120 (61.5)	13 (6.7)	62 (31.8)
Does your campus include sexual orientation in public written statements about diversity and multiculturalism?	Hetero	532 (49.4)	55 (5.1)	490 (45.5)
	LGBT	100 (51.3)	8 (4.1)	87 (44.6)
Does your campus prohibit discrimination based on gender identity or gender expression by including the words "gender identity" or "gender identity or expression" in its primary nondiscrimination statement or Equal Employment Opportunity policy?	Hetero	572 (53.1)	45 (4.2)	460 (42.7)
	LGBT	80 (41.0)	19 (9.7)	96 (49.3)
Does your campus offer health insurance coverage to employees’ same-sex partners?	Hetero	162 (15.0)	32 (3.0)	881 (82.0)
	LGBT	15 (7.8)	18 (9.3)	160 (82.9)
Does your campus include LGBT issues and concerns and/or representations of LGBT people in the following: grievance procedures, housing guidelines, admission application materials, health-care forms, alumni materials/publications?	Hetero	440 (40.9)	31 (2.9)	604 (56.2)
	LGBT	59 (30.4)	23 (11.9)	112 (57.7)
**LGBT Support and Institutional Commitment**				
Does your campus provide training for health-center staff to increase their sensitivity to the special healthcare needs of LGBT individuals?	Hetero	309 (28.7)	58 (5.4)	710 (65.9)
	LGBT	54 (28.0)	27 (14.0)	112 (58.0)
Does your campus have an LGBT concerns office or an LGBT student resource center (ie an institutionally funded space specifically for LGBT, gender, and sexuality education and/or support services)?	Hetero	277 (25.7)	193 (18.0)	604 (56.3)
	LGBT	25 (12.8)	80 (41.0)	90 (46.2)
Does your campus have a Safe Zone, Safe Space and/or Ally program (ie an ongoing network of visible people on campus who identify openly as allies/advocates for LGBT people and concerns)?	Hetero	487 (45.1)	98 (9.1)	494 (45.8)
	LGBT	95 (49.2)	28 (14.5)	70 (36.3)
**LGBT Academic Life**				
Does your campus integrate LGBT issues into existing courses when appropriate?	Hetero	633 (58.9)	99 (9.2)	343 (31.9)
	LGBT	113 (58.5)	39 (20.2)	41 (21.3)
Does your campus have a significant number of LGB-inclusive books and periodicals on sexual orientation topics in the campus library/libraries?	Hetero	109 (10.1)	99 (9.2)	869 (80.7)
	LGBT	20 (10.3)	40 (20.6)	134 (69.1)
**LGBT Student Life**				
Does your campus regularly offer activities and events to increase awareness of the experiences and concerns of lesbians, gay men, and bisexuals?	Hetero	520 (48.3)	178 (16.5)	379 (35.2)
	LGBT	78 (40.0)	57 (29.2)	60 (30.8)
Does your campus regularly hold social events specifically for LGBT students?	Hetero	259 (24.0)	296 (27.5)	523 (48.5)
	LGBT	37 (19.1)	83 (42.8)	74 (38.1)
Does your campus have a college/university-recognized organization for LGBT students and allies?	Hetero	582 (54.1)	109 (10.1)	385 (35.8)
	LGBT	104 (53.3)	40 (20.5)	51 (26.2)
Does your campus have any student organizations that primarily serve the needs of underrepresented and/or multicultural LGBT populations (eg LGBT Latinos/Latinas, international LGBT students, LGBT students with disabilities)?	Hetero	363 (33.7)	181 (16.8)	534 (49.5)
	LGBT	52 (26.8)	70 (36.1)	72 (37.1)
Does your campus have any student organizations that primarily serve the social and/or recreational needs of LGBT students (eg gay social fraternity, lesbian volleyball club, gay coed lacrosse club)?	Hetero	217 (20.2)	264 (24.6)	594 (55.2)
	LGBT	27 (14.0)	88 (45.6)	78 (40.4)
Does your campus have any academically focused LGBT student organizations (eg LGBT Medical Association, LGBT Public Relations Organization, Out Lawyers Association)?	Hetero	270 (25.1)	216 (20.1)	591 (54.8)
	LGBT	36 (18.7)	84 (43.5)	73 (37.8)
**LGBT Counseling and Health**				
Does your campus offer support groups for LGBT individuals in the process of coming out and for other LGBT issues/concerns?	Hetero	287 (26.7)	91 (8.5)	697 (64.8)
	LGBT	36 (18.5)	64 (32.8)	95 (48.7)
Does your campus offer individual counseling for students that is sensitive and affirming for (supportive) LGBT issues/concerns?	Hetero	497 (46.1)	37 (3.5)	543 (50.4)
	LGBT	93 (48.4)	21 (10.9)	78 (40.7)
Does your campus provide training for health-center staff to increase their sensitivity to the special healthcare needs of LGBT individuals?	Hetero	309 (28.7)	58 (5.4)	710 (65.9)
	LGBT	54 (28.0)	27 (14.0)	112 (58.0)

### Associations Between Survey Responses

A negative correlation was found between levels of perceived social support and levels of depression, where students with higher levels of perceived social support displayed lower levels of depression (*r* = -.33, *P <* .001). A positive correlation was found between comfort with disclosure of orientation and levels of depression, where students who reported higher discomfort with disclosure of orientation also had higher levels of depression (*r* = .22, *P <* .001). A slight negative correlation was found between comfort with disclosure of orientation and the campus climate (*r* = -.12, *P <* .001). Students who rated the campus as non-inclusive also reported discomfort with disclosure of their orientation.

## Discussion

The purpose of the current study was to investigate the relationship between sexual orientation and gender identity in regard to depression, levels of perceived social support, comfort with disclosure of orientation, and the LGBT campus climate in osteopathic medical schools. Results suggested that LGB students had higher levels of depression, slightly lower levels of perceived social support, and more discomfort with disclosure of orientation. A majority of students rated the campus climate as noninclusive. These results are consistent with other studies that showed LGB individuals were more likely to be exposed to negative interactions, including discrimination, harassment, isolation, and low social support [27,28].

In the current study, LGB students were 2.2 times more likely to be depressed then heterosexual students. Previous research findings show that LGBT individuals are at increased risk for psychosocial problems, including depression, suicide, and other psychiatric disorders [5-11]. Since research has suggested a link between psychosocial health and academic success [13,14], osteopathic medical schools should consider implementing support systems for LGBT students at the institutional level.

In general, the osteopathic medical students who responded to our survey had relatively high levels of perceived social support, regardless of sexual orientation. However, due to effect size, self-identified LGB students were more likely to indicate lower levels of perceived social support compared with heterosexual only students. Previous research has shown the importance of social support in the proper identity formation of LGB individuals and that those exposed to negative social interactions are much more likely to experience barriers in regard to self-acceptance [29-31]. Another study has suggested a strong link between low social support and psychosocial problems, such as depression, in minority populations [32]. In support of this previous research, our study also displayed a moderate correlation between social support and depression. This suggests the existence of a vulnerable subset within the population: LGB osteopathic medical students with low perceived social support. These students are likely to be at the highest risk for depression. However, contrary to these findings, our study seemed to indicate that even though LGB osteopathic medical students reported high levels of social support, they were still at an increased risk of being depressed. As such, it appears that regardless of perceived levels of social support, LGB osteopathic medical students display decreased psychosocial health. This is of practical importance since previous research has suggested a link between psychosocial health and academic success [13,14]. These findings suggest that the relationship between depression, social support and minority status in osteopathic medical students may be mitigated by other factors; future research is needed to examine this area more closely.

In the current study, LGB students were 3.9 times more likely to report discomfort with disclosure of orientation compared with heterosexual only students. These findings support results from previous research [18,33]. For example, Rankin reported that 60% of lesbian, gay, and bisexual undergraduate students remained closeted due to fear of discrimination [33]. In studies of medical students, many LGB students reported discomfort with being open about their sexual orientation [5] and an overwhelming majority did not disclose their orientation during the application process [18]. In the current study, there was a correlation between comfort with disclosure of orientation and depression, where students who reported higher discomfort also had higher levels of depression. Perhaps implementing an inclusive environment at medical schools, in which diversity is embraced, may address this problem.

LGB students were more likely to rate the campus climate as noninclusive in regard to LGBT issues, and those who rated the campus as noninclusive also reported discomfort with disclosure of their orientation. In a study by Rankin, almost 74% of lesbian, gay, and bisexual students rated their campus as noninclusive [32]. Other studies have shown that students who viewed their campus as more hostile in regard to LGBT issues were more likely to not disclose their sexual orientation [18,33].

Finally, due to the lack of transgender respondents, no comparisons could be made in regard to this population. This further highlights the invisibility of the transgender population in both medical education and research. The potential cause of this invisibility, be it due to underrepresentation in the osteopathic medical profession or discomfort with disclosure, should be examined in future research.

Results from the current study point to the important role that the campus culture can play during osteopathic medical training. The campus culture consists of the overarching environment that students are exposed to on a day-to-day basis and a positive culture would involve having LGBT-friendly institutional policies, grievance procedures, and equal opportunity measures. Osteopathic medical schools should considering creating a more LGBT-inclusive campus environment. Improving the campus climate may be one way of addressing LGB students’ significantly higher levels of depression and discomfort with disclosure of orientation. Some suggestions for improving the campus climate include: having a non-discrimination policy/statement that includes *sex, sexual orientation and gender expression;* offering insurance coverage to employees’ same-sex partners; the presence of a professional staff member who promotes diversity and increases campus awareness (including LGBT) as part of their job description.

The current study had several limitations. The sample contained students from only 6 osteopathic medical schools, making the results limited to the particular geographic locations of each of the schools. In general, osteopathic medical schools from the southern and eastern regions of the United States were underrepresented, potentially skewing the study results. Further, previous research has suggested that social desirability bias often limits the disclosure of negative views, such as depression or low social support [34]. This reluctance to disclose negative views may explain the high percentage of respondents in the current study who indicated high social support.

## Conclusions

Overall, the current study indicated that a relationship existed between sexual orientation and levels of depression, perceived social support, comfort with disclosure of orientation, and the LGBT campus climate in osteopathic students. Based on these results, osteopathic medical schools should consider closely examining their campus culture, in order to ensure that a positive and inclusive environment exists for all students. By embracing diversity in osteopathic medical education, it may be possible to create multicultural physicians who can competently and compassionately practice osteopathic medicine.

## Competing interests

The authors declare that they have no competing interests.

## Authors’ contributions

JL conceived the study, participated in its design and coordination, performed the statistical analysis and helped to draft the manuscript. TS participated in its design and coordination and helped to draft the manuscript. Both authors read and approved the final manuscript.

## Pre-publication history

The pre-publication history for this paper can be accessed here:

http://www.biomedcentral.com/1472-6920/14/171/prepub
